# Supplementary material for: Analysis of PPARγ Signaling Activity in Psoriasis
Source: Int J Mol Sci. 2021 Aug 10;22(16):8603. doi: 10.3390/ijms22168603 (PMC8395241; doi:10.3390/ijms22168603)
Supplement: Supplementary file 1 [file ijms-22-08603-s001.zip › Supplemental materials_Analysis of PPARg signaling activity in psoriasis/Pathway models/Models images and html files/Interleukin-17 and interleukin-22 signaling in psoriasis/2199.html]

CEBPA


# Protein CEBPA

|  |  |
| --- | --- |
| URN | urn:agi-llid:1050 |
| Total Entities | 0 |
| Connectivity | 4535 |
| Name | CEBPA |
| Description | CCAAT/enhancer binding protein (C/EBP), alpha |
| Notes | This intronless gene encodes a transcription factor that contains a basic leucine zipper (bZIP) domain and recognizes the CCAAT motif in the promoters of target genes. The encoded protein functions in homodimers and also heterodimers with CCAAT/enhancer-binding proteins beta and gamma. Activity of this protein can modulate the expression of genes involved in cell cycle regulation as well as in body weight homeostasis. Mutation of this gene is associated with acute myeloid leukemia. The use of alternative in-frame non-AUG (GUG) and AUG start codons results in protein isoforms with different lengths. Differential translation initiation is mediated by an out-of-frame, upstream open reading frame which is located between the GUG and the first AUG start codons. [provided by RefSeq, Dec 2013] |
| Primary Cell Localization | Nucleus |
| Class | Transcription factor |

---

|  |  |
| --- | --- |
| Pathway | Androgen Deficiency in Male Obesity |
|  | Androgens in Adipocyte Activation |
|  | Keep out Signal: LTF Inhibits Neutrophil Recruitment and Inflammation |
|  | Eosinophil Activation |
|  | Mast-Cell Activation via IgE Signaling |
|  | Negative Acute Phase Proteins Synthesis |
|  | Adipokines Production by Adipocyte |
|  | Hematopoietic Cell Lineage: B-cell (mouse) |
|  | Androgens in Sebocyte Maturation |
|  | IL22 Induces Keratinocyte Proliferation in Psoriasis |
|  | Adiponectin Synthesis Declines in Insulin Resistance |
|  | Gluconeogenesis Impairment in non-Alcoholic Fatty Liver Disease |
|  | PNPLA3 in non-Alcoholic Fatty Liver Disease |
|  | Lipid Metabolism Impairement in non-Alcoholic Fatty Liver Disease |
|  | Graves Ophthalmopathy |
|  | Adipocyte Hypertrophy and Hyperplasia |
|  | Adipokines Production by Adipocyte Impaired in Obesity |
|  | Proteins Involved in Obesity |
|  | Metastatic Colorectal Cancer |
|  | Proteins Involved in Acute Myeloid Leukemia |
|  | RARA Signaling in Acute Myeloid Leukemia (M3) |
|  | Acute Myeloid Leukemia |
|  | Myeloid Cell Differentiation Block in Leukemia |
|  | Chronic Myeloid Leukemia |
|  | Proteins Involved in Chronic Myeloid Leukemia |
|  | Eosinophil Activation and Degranulation in Asthma |
|  | Club-Cell Role in Asthma |
|  | Mast-Cell Activation in Asthma |
|  | Proteins Involved in Polycystic Ovary Syndrome |
|  | Li-Fraumeni Syndrome |
|  | Proteins Involved in Huntington Disease |
|  | Proteins Involved in Endometriosis |
|  | Fatty Acid Synthase (FASN) Signaling |
|  | Prostate Cancer |
|  | Proteins Involved in Prostate Cancer |
|  | Proteins Involved in Polycystic Kidney Disease |
|  | Elevated Receptors -> Expression Targets in Adipose Tissue |
|  | CCL3 Expression Targets |
|  | EGF -> CTNN Expression Targets |
|  | EGF -> AP-1/ATF Expression Targets |
|  | EGF -> STAT Expression Targets |
|  | Elevated Receptors -> Expression Targets in Gallbladder |
|  | GH1/GHR -> NF-kB/ELK/SRF/MYC Expression Targets |
|  | GH1/GHR -> STAT Expression Targets |
|  | TERT Activation in Cancer |
|  | Cancer Cells Inhibit Adipocyte Differentiation |
|  | IGF1 -> CEBPA/FOXO1A Expression Targets |
|  | IGF2 -> CEBPA/FOXO1A Expression Targets |
|  | Insulin -> CEBPA/CTNNB/FOXA/FOXO Expression Targets |
|  | Insulin -> MEF/MYOD Expression Targets |
|  | Insulin -> ELK/SRF/HIF1A/MYC/SREBF Expression Targets |
|  | IGF1R -> CEBPA/FOXO1A Signaling |
|  | IL6 Expression Targets |
|  | IL11 Expression Targets |
|  | Prostaglandin F Expression Targets |
|  | NOTCH -> Expression Targets in Thymus |
|  | OSM/OSMR Expression Targets |
|  | EDN1 Expression Targets |
|  | CSF1 -> STAT Expression Targets |
|  | CSF1 -> AP-1/CREB/CREBBP/MYC Expression Targets |
|  | TGFB1-TGFBR1/AP-1 Expression Targets |
|  | TGFB1-ACVRL1 Expression Targets |
|  | TLR4 -> AP-1/EGR1/HIF1A Expression Targets |
|  | IL1B Expression Targets |
|  | IL1A Expression Targets |
|  | Figure 3\_Mixed network of dexamethasone targets |
|  | Figure 3\_Mixed network of dexamethasone targets |
|  | 2\_Interleukin-17 and interleukin-22 signaling in psoriasis |
|  | 2\_The role of endothelial cells in pyelonephritis |
|  | 2\_Impaired steroidogenesis in PCOS |
|  | 2\_2\_Eosinophilia and neutrophilia in asthma: eosinophil apoptosis |
|  | 1\_Triglyceride storage in NAFLD |
|  | Interleukin-17 and interleukin-22 signaling in psoriasis |

---

|  |  |
| --- | --- |
| Group | Genes with Mutations Associated with Acute Myeloid Leukemia |
|  | Proteins Involved in Li-Fraumeni Syndrome |

---

|  |  |
| --- | --- |
| MedScan ID | 1050 |

---

|  |  |
| --- | --- |
| LocusLink ID | 1050 |
|  | 12606 |
|  | 24252 |

---

|  |  |
| --- | --- |
| Alias | C/EBPa |
|  | CCAAT/enhancer binding protein alpha |
|  | LOC51011 |
|  | CEBPA |
|  | CCAAT/enhancer-binding protein |
|  | DBPCEP |
|  | CCAAT/enhancer binding protein (C/EBP), alpha |
|  | OTTHUMP00000220269 |
|  | CCAAT/enhancer binding protein |
|  | CAAT/enhancer binding protein alpha |
|  | C/EBP |
|  | transcription factor C EBP alpha |
|  | CEBP |
|  | CAAT/enhancer-binding protein, DNA-binding protein |
|  | CGI-105S |
|  | C/EBP-alpha |
|  | CCAAT/enhancer-binding protein alpha |
|  | CBF-A |
|  | C/ebpalpha |
|  | C/EBP alpha |
|  | CAAT/enhancer-binding protein DNA-binding protein |
|  | CCAAT/enhancer binding protein, alpha |

---

|  |  |
| --- | --- |
| GO ID | 0003677 |
|  | 0001228 |
|  | 0003700 |
|  | 0000981 |
|  | 0000978 |
|  | 0019900 |
|  | 0042803 |
|  | 0003713 |
|  | 0008134 |
|  | 0044212 |
|  | 0007219 |
|  | 0050873 |
|  | 0048469 |
|  | 0071285 |
|  | 0071407 |
|  | 0071356 |
|  | 0008203 |
|  | 0019221 |
|  | 0001892 |
|  | 0045444 |
|  | 0006091 |
|  | 0042593 |
|  | 0030851 |
|  | 0048839 |
|  | 0070102 |
|  | 0055088 |
|  | 0001889 |
|  | 0030324 |
|  | 0030225 |
|  | 0007005 |
|  | 0030099 |
|  | 0008285 |
|  | 0045736 |
|  | 0000122 |
|  | 0045892 |
|  | 0045600 |
|  | 0050729 |
|  | 0043032 |
|  | 0045669 |
|  | 0032436 |
|  | 0045944 |
|  | 0045945 |
|  | 0006366 |
|  | 0006351 |
|  | 0000050 |
|  | 0016032 |
|  | 0050872 |
|  | 0090575 |
|  | 0043231 |
|  | 0005730 |
|  | 0005654 |
|  | 0005634 |
|  | 0005667 |
|  | 0071837 |
|  | 0001013 |
|  | 0042826 |
|  | 0019904 |
|  | 0046982 |
|  | 0044877 |
|  | 0043565 |
|  | 0007275 |
|  | 0045786 |
|  | 2000144 |
|  | 0045893 |
|  | 0042127 |
|  | 0006357 |
|  | 0006355 |
|  | 0006360 |
|  | 0036488 |
|  | 0035189 |
|  | 0016363 |
|  | 0032991 |
|  | 0006953 |
|  | 0031100 |
|  | 0030154 |
|  | 0035690 |
|  | 0007613 |
|  | 0002076 |
|  | 0071548 |
|  | 0007584 |
|  | 0080184 |
|  | 0033274 |
|  | 0003705 |
|  | 0001077 |
|  | 0032403 |
|  | 0043234 |
|  | 0051384 |
|  | 0019048 |
|  | 0010843 |
|  | 0003704 |
|  | 0044419 |
|  | 0010552 |
|  | 0016563 |
|  | 0016564 |

---

|  |  |
| --- | --- |
| KEGG ID | hsa:1050 |
|  | mmu:12606 |
|  | rno:24252 |

---

|  |  |
| --- | --- |
| Organism | Homo sapiens {Organism urn:agi-taxid:9606} |
|  | Mus musculus {Organism urn:agi-taxid:10090} |
|  | Rattus norvegicus {Organism urn:agi-taxid:10116} |
|  | Homo sapiens |
|  | Mus musculus |
|  | Rattus norvegicus |

---

|  |  |
| --- | --- |
| Mouse chromosome position | 7 21.02 cM |
|  | 7 12.0 cM |

---

|  |  |
| --- | --- |
| OMIM ID | 116897 |
|  | 601626 |

---

|  |  |
| --- | --- |
| Rat chromosome position | 1q21 |
|  | 1q22 |

---

|  |  |
| --- | --- |
| Hugo ID | 1833 |
|  | HGNC:1833 |

---

|  |  |
| --- | --- |
| Human chromosome position | 19q13.11 |
|  | 19q13.1 |

---

|  |  |
| --- | --- |
| Swiss-Prot Accession | P49715 |
|  | P49715.3 |
|  | P53566 |
|  | P53566.2 |
|  | P05554 |
|  | P05554.3 |
|  | A7LNP2 |
|  | P78319 |
|  | Q05CA4 |
|  | Q91XB6 |
|  | Q6P3S4 |
|  | Q3U2H8 |
|  | Q9QWK8 |

---

|  |  |
| --- | --- |
| PIR ID | JC4311 |
|  | I49575 |
|  | S06890 |

---

|  |  |
| --- | --- |
| GenBank ID | NC\_000019 |
|  | NM\_004364 |
|  | NP\_004355 |
|  | NM\_001285829 |
|  | NP\_001272758 |
|  | NM\_001287435 |
|  | NP\_001274364 |
|  | NM\_001287424 |
|  | NP\_001274353 |
|  | NG\_012022 |
|  | AC008738 |
|  | EU048234 |
|  | ABS82765 |
|  | S75265 |
|  | AAB33475 |
|  | U34070 |
|  | AAC50235 |
|  | BC027902 |
|  | AAH27902 |
|  | BC063874 |
|  | AAH63874 |
|  | X87248 |
|  | CAA60698 |
|  | Y11525 |
|  | CAA72289 |
|  | P49715 |
|  | NC\_000073 |
|  | NM\_007678 |
|  | NP\_031704 |
|  | NM\_001287514 |
|  | NP\_001274443 |
|  | NM\_001287515 |
|  | NP\_001274444 |
|  | NM\_001287521 |
|  | NP\_001274450 |
|  | NM\_001287523 |
|  | NP\_001274452 |
|  | AC150683 |
|  | CH466618 |
|  | EDL03027 |
|  | M62362 |
|  | AAA37374 |
|  | AK155281 |
|  | BAE33162 |
|  | AK160443 |
|  | AW045436 |
|  | BC011118 |
|  | AAH11118 |
|  | BC028890 |
|  | AAH28890 |
|  | BC051102 |
|  | AAH51102 |
|  | BC058161 |
|  | AAH58161 |
|  | BC106174 |
|  | P53566 |
|  | NC\_005100 |
|  | NM\_001287577 |
|  | NP\_001274506 |
|  | NM\_012524 |
|  | NP\_036656 |
|  | NM\_001287578 |
|  | NP\_001274507 |
|  | NM\_001287579 |
|  | NP\_001274508 |
|  | AC\_000069 |
|  | AAHX01004671 |
|  | AB020756 |
|  | BAA36342 |
|  | AC109741 |
|  | CH473979 |
|  | EDM07633 |
|  | X12752 |
|  | CAA31242 |
|  | P05554 |
|  | NC\_018930 |
|  | AMYH02035938 |
|  | BC160133 |
|  | AAI60133 |
|  | AC\_000029 |
|  | AAHY01060488 |
|  | AC\_000151 |
|  | ABBA01012597 |
|  | ABBA01012598 |
|  | AABR06004416 |
|  | NT\_011109 |
|  | NW\_927206 |
|  | NW\_001838492 |
|  | AC\_000062 |
|  | Q6P3S4 |
|  | NT\_039413 |
|  | NW\_001030843 |
|  | Q3U2H8 |
|  | NW\_001084763 |
|  | NW\_047557 |
|  | Q9QWK8 |

---

|  |  |
| --- | --- |
| Swiss-Prot ID | CEBPA\_HUMAN |
|  | CEBPA\_MOUSE |
|  | CEBPA\_RAT |

---

|  |  |
| --- | --- |
| Cell Localization | Nucleus |
|  | Nucleolus |
|  | Nucleus, nucleolus |

---

|  |  |
| --- | --- |
| Ensembl ID | ENSG00000245848 |
|  | ENSP00000427514.1 |
|  | ENST00000498907.3 |
|  | ENSMUSG00000034957 |
|  | ENSMUSP00000096129.4 |
|  | ENSMUST00000042985.10 |
|  | ENSRNOG00000010918 |
|  | ENSRNOP00000063123.2 |
|  | ENSRNOT00000014517.5 |
|  | ENSP00000427514 |
|  | ENST00000498907 |
|  | ENSMUSP00000096129 |
|  | ENSMUST00000042985 |
|  | ENSRNOP00000063123 |
|  | ENSRNOT00000014517 |

---

|  |  |
| --- | --- |
| MGI ID | MGI:99480 |
|  | 99480 |

---

|  |  |
| --- | --- |
| RGD ID | 2326 |

---

|  |  |
| --- | --- |
| Unigene ID | Hs.76171 |
|  | Mm.349667 |
|  | Hs.740432 |
|  | Rn.204833 |

---

|  |  |
| --- | --- |
| Homologene ID | 3211 |

---

|  |  |
| --- | --- |
| Shape | O-vertex |

---

|  |  |
| --- | --- |
| IPI ID | IPI00132435 |
|  | IPI00200620 |
|  | IPI00292025 |
|  | IPI00383649 |

---
